# Potassium dynamics in sickle cell anemia: clinical implications and pathophysiological insights

**DOI:** 10.1097/MS9.0000000000002551

**Published:** 2024-09-10

**Authors:** Emmanuel Ifeanyi Obeagu

**Affiliations:** Department of Medical Laboratory Science, Kampala International University, Ishaka, Uganda

**Keywords:** cell dehydration, hemolytic anemia, potassium dynamics, sickle cell anemia, vaso-occlusion

## Abstract

Potassium dynamics are critical in the pathophysiology of sickle cell anemia (SCA), a genetic disorder characterized by the presence of abnormally shaped red blood cells that lead to various complications such as vaso-occlusive crises and hemolytic anemia. This review focuses on the clinical implications and pathophysiological insights of potassium regulation in SCA, highlighting its impact on disease progression and potential therapeutic strategies. The dysregulation of potassium transport in SCA leads to significant K+ efflux and cellular dehydration, exacerbating the sickling process. Dehydrated sickle cells, due to potassium loss, become more rigid and prone to causing blockages in small blood vessels, leading to painful vaso-occlusive crises and ischemia. Furthermore, chronic hemolysis in SCA, aggravated by potassium imbalance, contributes to severe anemia and systemic complications. These insights underscore the importance of maintaining potassium homeostasis to mitigate disease severity and improve patient outcomes. Therapeutic strategies targeting potassium regulation show promise in managing SCA. Inhibitors of the Gardos channel, such as senicapoc, have demonstrated potential in reducing sickling and hemolysis. Additionally, hydration therapy plays a crucial role in maintaining electrolyte balance and preventing RBC dehydration. A comprehensive approach that includes monitoring and correcting electrolyte imbalances, along with standard treatments like hydroxyurea and blood transfusions, is essential for effective disease management.

## Introduction

Highlights

- Molecular pathways: Elucidating hemoglobin polymerization and RBC sickling mechanisms.
- Targeted therapies: Advancing pharmacological agents and gene therapies.
- Early intervention: Promoting early diagnosis and personalized care.
- Psychosocial support: Integrating emotional and educational resources.
- Collaborative research: Encouraging interdisciplinary and industry partnerships.

Sickle cell anemia (SCA) is a hereditary hemoglobinopathy resulting from a single nucleotide mutation in the β-globin gene, leading to the production of abnormal hemoglobin S (HbS). This genetic alteration causes red blood cells (RBCs) to adopt a sickle shape under hypoxic conditions, which impairs their function and lifespan. The distorted shape and reduced flexibility of sickle cells contribute to vaso-occlusive events, hemolysis, and chronic anemia, manifesting in severe clinical complications, and reduced life expectancy^1–3^. The pathophysiology of SCA is complex and multifactorial, involving a cascade of cellular and molecular events^4^. Central to this process is the polymerization of HbS, which occurs when deoxygenated, leading to the formation of rigid, sickle-shaped RBCs^5^. These cells obstruct blood flow in small vessels, causing ischemia and tissue damage. Hemolysis, both intravascular and extravascular, is another hallmark of SCA, resulting in chronic anemia and a range of associated symptoms, including fatigue, jaundice, and increased susceptibility to infections^6,7^. Potassium (K+) homeostasis is crucial for maintaining cellular function and integrity^8^. In RBCs, potassium balance is particularly important for regulating cell volume and preventing dehydration. Dysregulation of potassium transport mechanisms can lead to significant cellular dehydration, exacerbating the sickling process. The role of potassium in SCA, therefore, extends beyond simple electrolyte balance, influencing the severity and progression of the disease.

Several key transport mechanisms regulate potassium levels in RBCs, including the K-Cl cotransporter, Gardos channel, and Na-K-ATPase pump^9^. The K-Cl cotransporter mediates the coupled movement of K+ and Cl- out of the cell, while the Gardos channel, a Ca2+-activated K+ channel, plays a critical role in K+ efflux during cellular dehydration. The Na-K-ATPase pump, meanwhile, actively maintains the gradient of Na+ and K+ across the cell membrane, essential for cellular homeostasis. In SCA, these potassium transport mechanisms often become dysregulated, leading to increased K+ efflux and cellular dehydration^10^. As water follows K+ out of the cell osmotically, sickle RBCs become more dehydrated, increasing intracellular HbS concentration and promoting further polymerization. Dehydrated sickle cells are more rigid and prone to adhere to the vascular endothelium, contributing to vaso-occlusive events and tissue ischemia. The clinical implications of potassium dysregulation in SCA are profound. Vaso-occlusive crises, a primary cause of morbidity and mortality in SCA, are closely linked to the dehydration and rigidity of sickle cells. Additionally, chronic hemolysis exacerbated by potassium imbalance leads to persistent anemia and systemic complications^11^. Vaso-occlusive crises are a defining feature of SCA, caused by the obstruction of small blood vessels by sickled RBCs. Potassium dynamics significantly influence these crises, as dehydrated and rigid RBCs are more likely to cause vascular blockages. This underscores the importance of maintaining potassium homeostasis to reduce the frequency and severity of vaso-occlusive events, thereby improving the quality of life for SCA patients^12^. Chronic hemolysis in SCA leads to anemia, contributing to symptoms such as fatigue, pallor, and jaundice. Potassium loss from RBCs exacerbates hemolysis by increasing cell fragility and promoting sickling. Therapeutic approaches that stabilize potassium levels within RBCs may help reduce hemolysis, thereby ameliorating anemia and its associated symptoms. This highlights the potential of targeted interventions in improving hemolytic outcomes in SCA^13^. Several therapeutic strategies aim to address potassium dysregulation in SCA. Gardos channel inhibitors, such as senicapoc, have shown promise in reducing RBC dehydration and sickling. Additionally, hydration therapy, which ensures adequate fluid intake and electrolyte balance, is crucial for preventing cellular dehydration and reducing vaso-occlusive crises. A holistic approach that includes these strategies, alongside standard treatments like hydroxyurea and blood transfusions, is essential for effective SCA management.

### Aim

The aim of understanding the pathophysiological insights into sickle cell anemia (SCA) is to elucidate the complex mechanisms underlying its clinical manifestations and complications.

### Rationale

The rationale for studying the pathophysiological insights into sickle cell anemia (SCA) lies in the complexity and severity of this genetic disorder, which affects millions of people globally. SCA presents with a wide range of clinical symptoms, including recurrent pain crises (vaso-occlusive crises), chronic anemia, and increased susceptibility to infections. Understanding the pathophysiological basis of these symptoms helps in developing targeted therapies to alleviate suffering and improve quality of life. Individuals with SCA are at risk of developing severe complications such as stroke, acute chest syndrome, renal dysfunction, and pulmonary hypertension. Exploring the mechanisms underlying these complications provides insights into preventive strategies and early interventions to mitigate long-term organ damage. SCA is caused by a single point mutation in the β-globin gene, resulting in the production of abnormal hemoglobin (HbS). This genetic alteration leads to the polymerization of HbS under conditions of low oxygen tension, triggering a cascade of events that culminate in RBC sickling, hemolysis, and vascular occlusion. Understanding these genetic and molecular pathways facilitates the development of targeted therapies, such as gene editing and gene therapy, aimed at correcting the underlying genetic defect. SCA disproportionately affects populations of African, Mediterranean, Middle Eastern, and Indian ancestry, leading to significant healthcare disparities. Studying the pathophysiology of SCA informs public health strategies aimed at improving access to comprehensive care, reducing healthcare costs associated with acute complications, and promoting early diagnosis through newborn screening programs. Advances in understanding the pathophysiology of SCA pave the way for innovative treatment modalities and research initiatives. This includes the development of disease-modifying therapies like hydroxyurea, novel agents targeting specific pathways (e.g. Gardos channel inhibitors), and emerging gene therapies that hold promise for potentially curative approaches.

## Review methodology

### Search strategy

A systematic approach was adopted to identify relevant studies and articles pertaining to the pathophysiology of SCA. Electronic databases, including PubMed, MEDLINE, and Google Scholar, were searched using keywords such as ‘sickle cell anemia’, ‘pathophysiology’, ‘hemoglobin S’, ‘vascular occlusion’, and ‘chronic hemolysis’. The search was limited to articles published in peer-reviewed journals within the last 10 years, with a focus on original research, reviews, and meta-analyses.

### Selection criteria

Articles were screened based on predefined inclusion criteria. Studies were included if they provided insights into the genetic basis of SCA, molecular mechanisms of hemoglobin polymerization, pathophysiological pathways leading to vaso-occlusive crises (VOC), chronic hemolysis, and associated complications such as stroke, acute chest syndrome, and renal dysfunction. Non-English language articles and studies lacking relevance to the primary objectives were excluded to ensure the quality and relevance of the review.

### Data extraction and synthesis

Data extraction focused on key findings related to the pathophysiological mechanisms underlying SCA. Emphasis was placed on identifying molecular pathways involved in HbS polymerization, factors contributing to RBC sickling and adherence, endothelial dysfunction, and inflammatory responses. Data synthesis involved categorizing and summarizing findings to elucidate the sequence of events from genetic mutation to clinical outcomes, highlighting gaps in current knowledge and potential implications for therapeutic interventions.

### Quality assessment

The quality of included studies was assessed using established criteria for evaluating research methodology, study design, sample size, and statistical analysis. Studies with robust methodologies and findings supported by adequate data were prioritized in the synthesis of results to ensure the reliability and validity of conclusions drawn from the review.

## Potassium homeostasis in sickle cell anemia

### Potassium transport mechanisms

Potassium (K+) homeostasis in red blood cells (RBCs) is essential for maintaining cellular volume, integrity, and function. In sickle cell anemia (SCA), the regulation of potassium transport becomes particularly critical due to its impact on cell dehydration and sickling^14^. The K-Cl cotransporter mediates the coupled movement of potassium (K+) and chloride (Cl-) ions out of the RBC. This process is electroneutral, meaning that it does not generate an electric current, but it significantly influences cell volume by regulating the osmotic balance. In SCA, increased activity of the K-Cl cotransporter can lead to excessive K+ loss, contributing to cellular dehydration^15^. This dehydration promotes the polymerization of hemoglobin S (HbS) and the sickling of RBCs, exacerbating the clinical symptoms of the disease. The Gardos channel, also known as the Ca2+-activated K+ channel, is pivotal in the regulation of potassium efflux from RBCs^16^. When intracellular calcium (Ca2+) levels rise, the Gardos channel opens, allowing K+ to exit the cell. This efflux is accompanied by water loss, leading to cell shrinkage and increased intracellular HbS concentration. In SCA, the activation of the Gardos channel contributes significantly to the dehydration of sickle cells, enhancing their rigidity and propensity to block blood vessels. Inhibiting this channel has been proposed as a therapeutic strategy to reduce cell dehydration and sickling.

The Na-K-ATPase pump is a crucial membrane-bound enzyme that actively maintains the gradients of sodium (Na+) and potassium (K+) across the RBC membrane. It pumps three Na+ ions out of the cell and two K+ ions into the cell, using ATP as an energy source. This pump helps maintain the cell’s resting potential and osmotic balance. In SCA, the function of the Na-K-ATPase pump may be compromised, leading to disruptions in ion balance and contributing to cellular dysfunction and hemolysis^17^. Piezo1 channels are mechanosensitive ion channels that respond to mechanical stress and membrane tension by allowing the influx of cations, including K+. Recent studies suggest that these channels play a role in the regulation of RBC volume and could be involved in the pathophysiology of SCA^18^. The activation of Piezo1 channels in sickle cells may contribute to the dysregulation of ion homeostasis, promoting cell dehydration and sickling. The NKCC1 (Na+-K+-2Cl- cotransporter) is another important player in ion transport within RBCs. It facilitates the simultaneous uptake of Na+, K+, and Cl- ions into the cell. In SCA, alterations in NKCC1 activity could affect intracellular ion concentrations, influencing cell volume and hydration status^19^. One promising therapeutic strategy for SCA involves the inhibition of the Gardos channel. By preventing K+ efflux and subsequent water loss, Gardos channel inhibitors, such as senicapoc, aim to reduce RBC dehydration and sickling. Clinical trials have shown that Gardos channel inhibition can decrease the frequency of vaso-occlusive crises and improve overall RBC health in SCA patients. However, further research is needed to fully establish the long-term efficacy and safety of these inhibitors. The dysregulation of potassium transport mechanisms in SCA has profound clinical implications^20^. Dehydrated and rigid sickle cells are more likely to adhere to the vascular endothelium and cause blockages, leading to vaso-occlusive crises, ischemia, and organ damage. Chronic hemolysis, exacerbated by potassium imbalance, results in persistent anemia and systemic complications. Therefore, understanding and managing potassium transport is crucial for improving clinical outcomes in SCA patients.

### Impact on cell dehydration and sickling

In sickle cell anemia (SCA), the dehydration of red blood cells (RBCs) is a critical factor that exacerbates the sickling process^21^. Several mechanisms contribute to this dehydration, primarily involving the dysregulation of potassium (K+) transport. Key players include the Gardos channel, K-Cl cotransporter, and Na-K-ATPase pump. When the Gardos channel is activated by increased intracellular calcium (Ca2+) levels, it allows K+ to exit the cell. As K+ leaves the RBC, water follows osmotically, resulting in cell shrinkage and dehydration. The K-Cl cotransporter also contributes to this process by mediating the coupled movement of K+ and Cl- out of the cell, further promoting water loss. Dehydrated RBCs exhibit higher intracellular hemoglobin S (HbS) concentrations, which significantly increase the likelihood of HbS polymerization^20^. This polymerization process leads to the formation of rigid, sickle-shaped cells that are less deformable and more prone to hemolysis. The rigidity of dehydrated sickle cells also makes them more likely to adhere to the vascular endothelium, causing blockages in small blood vessels. These blockages result in vaso-occlusive crises, a hallmark of SCA characterized by severe pain, tissue ischemia, and organ damage. Potassium dynamics play a crucial role in the dehydration of RBCs in SCA^19^. The loss of K+ through the Gardos channel and K-Cl cotransporter leads to a significant reduction in cell volume. This process not only increases HbS concentration but also creates an environment that favors sickling. The Na-K-ATPase pump, which actively maintains K+ gradients across the cell membrane, may also be compromised in SCA, further contributing to ion imbalance and cellular dehydration. Dysregulated potassium transport thus directly impacts the physical properties of RBCs, promoting sickling and its associated complications.

The dehydration and subsequent sickling of RBCs are central to the occurrence of vaso-occlusive crises. These crises arise when rigid, dehydrated sickle cells obstruct capillaries and small blood vessels, impeding blood flow and causing acute pain episodes. The frequency and severity of these crises are closely linked to the extent of RBC dehydration. By maintaining proper potassium balance and preventing cell dehydration, it may be possible to reduce the incidence of vaso-occlusive crises and improve patient outcomes in SCA^20^. Chronic hemolysis is another significant consequence of RBC dehydration in SCA. Dehydrated sickle cells are more fragile and susceptible to rupture as they traverse the circulatory system. This ongoing destruction of RBCs leads to hemolytic anemia, characterized by low hemoglobin levels, fatigue, and jaundice. Potassium loss exacerbates hemolysis by increasing RBC fragility. Therapeutic strategies that stabilize potassium levels and prevent dehydration can help mitigate hemolysis and its associated symptoms^19^. The cumulative effects of vaso-occlusion and hemolysis contribute to long-term organ damage in SCA patients. Dehydrated, sickled cells can cause repeated episodes of ischemia-reperfusion injury in organs such as the spleen, kidneys, lungs, and brain. Over time, this can lead to chronic organ dysfunction, significantly impacting the patient’s quality of life and life expectancy. Proper management of potassium dynamics and RBC hydration is crucial to preventing organ damage and improving long-term outcomes^22–24^. Gardos channel inhibitors, such as senicapoc, have shown promise in reducing RBC dehydration and preventing sickling. Additionally, hydration therapy, aimed at maintaining adequate fluid intake and electrolyte balance, is essential for minimizing cell dehydration. By focusing on these therapeutic strategies, it is possible to address the underlying pathophysiological mechanisms of SCA and improve patient management.

### Pathophysiological insights

Pathophysiological insights into sickle cell anemia (SCA) reveal a complex interplay of genetic, molecular, and physiological factors that underlie the clinical manifestations and complications of this inherited hemoglobinopathy^25^. SCA is caused by a point mutation in the β-globin gene, leading to the production of abnormal hemoglobin known as hemoglobin S (HbS)^26^. Under conditions of low oxygen tension, HbS molecules polymerize and aggregate within red blood cells (RBCs), causing them to assume a rigid, sickle shape. This polymerization process is central to the pathophysiology of SCA, as sickled RBCs are less deformable and more prone to hemolysis and vascular occlusion. Sickled RBCs adhere to vascular endothelium and to each other, leading to the obstruction of small blood vessels and impaired blood flow^27^. This process, known as vaso-occlusion, contributes to tissue ischemia and the characteristic episodic pain crises seen in SCA. Endothelial dysfunction and activation of adhesion molecules further exacerbate microvascular occlusion, perpetuating tissue damage and inflammation. Episodes of vaso-occlusion followed by reperfusion lead to oxidative stress, inflammatory responses, and tissue injury. Ischemia-reperfusion injury contributes to organ damage, particularly in organs with high metabolic demands such as the brain, kidneys, and lungs. This cycle of injury and repair plays a significant role in the chronic complications associated with SCA, including organ dysfunction and progressive damage over time.

Sickled RBCs have a shortened lifespan due to their fragility and susceptibility to hemolysis. Chronic hemolysis results in the release of free hemoglobin into the plasma, leading to scavenging of nitric oxide (NO) and subsequent endothelial dysfunction. This imbalance in NO bioavailability contributes to vasoconstriction, further exacerbating vascular complications in SCA^28^. Alterations in red cell membrane structure and function are observed in SCA, including increased permeability to cations such as potassium (K+) and calcium (Ca2+). Dysregulation of ion transport systems, including the Gardos channel (KCa3.1), contributes to RBC dehydration and sickling. Sickled RBCs and damaged endothelial cells release inflammatory mediators, cytokines, and cell adhesion molecules that activate leukocytes and amplify the inflammatory response. Persistent inflammation contributes to a prothrombotic state, endothelial dysfunction, and the perpetuation of vaso-occlusive events. Functional asplenia, resulting from repeated vaso-occlusive events in the spleen, increases susceptibility to bacterial infections, particularly from encapsulated organisms. Reduced clearance of opsonized bacteria and impaired adaptive immune responses further compound infection risks in individuals with SCA.

## Clinical implications

### Vaso-occlusive crises

Vaso-occlusive crises (VOC) are a hallmark of sickle cell anemia (SCA), representing one of the most severe and debilitating complications of the disease. These crises occur when rigid, sickle-shaped red blood cells (RBCs) obstruct small blood vessels, leading to ischemia, tissue injury, and intense pain. The pathophysiology of VOC is multifactorial, involving the interaction of sickle cells with the vascular endothelium, inflammatory responses, and altered blood flow dynamics. The dehydration of RBCs, primarily due to dysregulated potassium (K+) transport, significantly exacerbates these processes^1^. RBC dehydration plays a crucial role in the pathogenesis of VOC. Dehydrated sickle cells have increased intracellular hemoglobin S (HbS) concentrations, which promote polymerization and the formation of rigid, sickle-shaped cells. These cells are less deformable and more prone to adhering to the vascular endothelium. When dehydrated sickle cells obstruct capillaries and small vessels, they impede blood flow, leading to localized hypoxia, inflammation, and pain. Maintaining potassium balance and preventing RBC dehydration are therefore critical strategies for reducing the frequency and severity of VOC^2^. Potassium transport mechanisms, such as the Gardos channel, K-Cl cotransporter, and Na-K-ATPase pump, are central to RBC dehydration in SCA. The Gardos channel, in particular, is activated by increased intracellular calcium (Ca2+) levels, allowing K+ to exit the cell. This K+ efflux is followed by water loss, resulting in cell shrinkage and increased HbS concentration. Inhibiting the Gardos channel has been shown to reduce RBC dehydration and the incidence of VOC. Similarly, the K-Cl cotransporter and Na-K-ATPase pump also contribute to maintaining K+ homeostasis and cell volume, influencing the occurrence of VOC^19^.

The clinical manifestations of VOC are diverse, with pain being the most prominent symptom. Pain episodes can vary in intensity and duration, often requiring hospitalization and significant medical intervention. The pain typically occurs in the bones, joints, and abdomen but can affect any part of the body. In addition to pain, VOC can cause fever, swelling, and limited mobility in affected areas. Repeated episodes of VOC can lead to chronic pain syndromes and long-term damage to organs and tissues^20^. Inflammation plays a key role in the development and propagation of VOC. The interaction of sickle cells with the vascular endothelium triggers the release of proinflammatory cytokines and adhesion molecules, which further promote the adhesion of sickle cells and leukocytes to the endothelium. This inflammatory response exacerbates vascular occlusion and tissue ischemia. Therapeutic strategies targeting inflammation, such as NSAIDs and corticosteroids, are often used to manage the symptoms of VOC^21^. The recurrent nature of VOC can cause cumulative damage to various organs, including the spleen, kidneys, lungs, and brain. In the spleen, repeated infarctions can lead to functional asplenia, increasing the risk of infections. Renal damage due to repeated VOC can result in chronic kidney disease. Pulmonary complications, such as acute chest syndrome, are common and can be life-threatening. Cerebral infarctions can lead to strokes and long-term neurological deficits. Preventing VOC is therefore crucial for reducing organ damage and improving long-term outcomes in SCA patients^22^. Effective management of VOC involves both preventive and acute treatment strategies. Preventive measures include the use of hydroxyurea, which increases fetal hemoglobin (HbF) levels and reduces HbS polymerization. Blood transfusions are also used to reduce the proportion of sickle cells and prevent VOC. During acute VOC episodes, pain management is the primary focus, using a combination of NSAIDs, opioids, and hydration therapy to alleviate symptoms. Emerging therapies targeting the underlying pathophysiology of VOC, such as Gardos channel inhibitors and anti-inflammatory agents, offer additional avenues for intervention^23^. Hydration therapy is a cornerstone in the management of VOC. Adequate hydration helps maintain blood volume and reduces the viscosity of blood, which can help alleviate vascular occlusions. Hydration also helps maintain electrolyte balance, particularly potassium, reducing the dehydration of RBCs. Patients are encouraged to maintain high fluid intake and may receive intravenous fluids during acute VOC episodes to ensure adequate hydration.

### Hemolytic anemia

Hemolytic anemia is a prominent feature and a major contributor to morbidity in individuals with sickle cell anemia (SCA)^29^. It results from the premature destruction of red blood cells (RBCs), which occurs at an accelerated rate in SCA due to the abnormal sickle-shaped morphology of the cells. This condition leads to a chronic shortage of RBCs in circulation, causing symptoms such as fatigue, pallor, jaundice, and increased susceptibility to infections. Understanding the mechanisms and consequences of hemolytic anemia in SCA is crucial for effective management and improved patient outcomes. The primary mechanism of hemolysis in SCA stems from the polymerization of hemoglobin S (HbS) under conditions of low oxygen tension^30^. Deoxygenated HbS molecules aggregate and form rigid, sickle-shaped cells. These cells are less flexible and more prone to rupture as they traverse the microvasculature, leading to premature destruction and hemolysis. The sickling process causes structural damage to the RBC membrane, rendering the cells more fragile. This membrane damage increases the susceptibility of sickle cells to mechanical stress and hemolysis. Additionally, altered membrane permeability and ion transport, including potassium (K+) loss, further contribute to RBC instability and hemolysis. Hemolysis in SCA occurs via both intravascular and extravascular mechanisms. Intravascular hemolysis results from the rupture of sickled RBCs within the bloodstream, releasing hemoglobin into the plasma. This free hemoglobin can scavenge nitric oxide (NO), leading to vasoconstriction and endothelial dysfunction. Extravascular hemolysis occurs predominantly in the spleen and liver, where macrophages phagocytose and degrade damaged RBCs, resulting in the release of bilirubin and iron.

Chronic hemolysis leads to anemia, characterized by reduced hemoglobin levels and diminished oxygen-carrying capacity of the blood. Anemic symptoms include fatigue, weakness, pallor, and shortness of breath. Severe anemia may necessitate blood transfusions to maintain adequate oxygen delivery to tissues. The breakdown of hemoglobin during hemolysis releases bilirubin, a yellow pigment. Elevated levels of unconjugated bilirubin in the bloodstream can cause jaundice, characterized by yellowing of the skin and sclerae. Free hemoglobin released during intravascular hemolysis scavenges NO, reducing its bioavailability and promoting vasoconstriction. This endothelial dysfunction contributes to the pathogenesis of vaso-occlusive crises, acute chest syndrome, and other vascular complications in SCA^31^. Chronic hemolysis results in increased iron turnover, leading to iron overload in tissues such as the liver, heart, and endocrine organs. Iron overload can cause organ damage and contribute to complications such as heart failure and endocrine dysfunction. Hydroxyurea is a disease-modifying therapy that increases fetal hemoglobin (HbF) levels, which inhibit sickle hemoglobin polymerization and reduce hemolysis^32^. It has been shown to decrease the frequency of vaso-occlusive crises and acute chest syndrome, as well as improve overall survival in SCA patients. Regular blood transfusions are used to maintain hemoglobin levels and reduce the severity of anemia in patients with SCA. Transfusions also help suppress erythropoiesis and decrease the proportion of sickled RBCs in circulation. For patients receiving chronic transfusions, iron chelation therapy is essential to mitigate iron overload and prevent organ damage. Chelators such as deferoxamine, deferasirox, and deferiprone bind excess iron and facilitate its excretion from the body. Managing complications of hemolytic anemia, such as jaundice, gallstones, and leg ulcers, requires comprehensive supportive care. Regular monitoring of hemoglobin levels, iron status, and organ function is essential for optimizing patient outcomes.

### Organ damage

Organ damage is a serious consequence of sickle cell anemia (SCA), primarily resulting from recurrent vaso-occlusive crises (VOC), chronic hemolysis, and other complications associated with the disease^3^. The impact of SCA on various organs underscores the complex and multisystem nature of the disorder, necessitating comprehensive management strategies to mitigate long-term complications and improve patient outcomes. The spleen is particularly vulnerable in individuals with SCA due to repeated episodes of vaso-occlusion and infarction. Over time, these ischemic events can lead to functional asplenia, where the spleen loses its ability to filter blood and remove aged or damaged red blood cells. Asplenia increases the risk of infections, particularly from encapsulated bacteria such as Streptococcus pneumoniae, Haemophilus influenzae, and Neisseria meningitidis. Patients with SCA are often prophylactically treated with antibiotics and vaccinations to reduce the risk of overwhelming infections. The kidneys are another organ commonly affected in SCA, primarily due to hemodynamic changes, microvascular occlusions, and chronic hemolysis. Sickle cell nephropathy can manifest as various renal complications, including hematuria, proteinuria, impaired concentration ability, and ultimately, chronic kidney disease (CKD). The deposition of hemosiderin and iron overload from chronic transfusions can exacerbate renal dysfunction, leading to progressive loss of kidney function over time^4^.

Acute chest syndrome (ACS) is a severe complication of SCA that affects the lungs and is characterized by pulmonary vaso-occlusion, inflammation, and hypoxia. ACS often presents with symptoms such as chest pain, dyspnea, cough, and fever. It is a leading cause of hospitalization and mortality in patients with SCA, necessitating prompt diagnosis and aggressive management, including supportive care, antibiotics, and possibly blood transfusions. Neurological complications in SCA can result from both acute events, such as strokes due to cerebral infarctions, and chronic conditions, including cognitive deficits and silent cerebral infarcts. Vaso-occlusion in the cerebral vasculature can lead to ischemic strokes, hemorrhagic strokes, or transient ischemic attacks (TIAs), causing varying degrees of neurological impairment. Regular monitoring and early intervention are crucial to prevent and manage these potentially debilitating complications. The liver can be affected by chronic hemolysis and iron overload, leading to hepatomegaly, cholelithiasis (gallstones), and liver dysfunction. Iron deposition in the liver can progress to iron overload, causing fibrosis, cirrhosis, and potentially liver failure in severe cases. Regular monitoring of liver function and iron status, coupled with iron chelation therapy when indicated, is essential to prevent irreversible liver damage. Cardiovascular complications, including cardiomyopathy, pulmonary hypertension, and heart failure, are increasingly recognized in patients with SCA. Chronic anemia and hemolysis contribute to increased cardiac output and vascular resistance, predisposing individuals to develop pulmonary hypertension. Furthermore, iron overload and myocardial iron deposition can lead to cardiomyopathy and congestive heart failure, necessitating comprehensive cardiac monitoring and management. Managing organ damage in SCA requires a multidisciplinary approach focused on preventing complications, optimizing organ function, and improving quality of life. Strategies include disease-modifying therapies such as hydroxyurea to reduce sickling, blood transfusions to alleviate anemia and prevent stroke, and supportive care to manage pain and other symptoms. Advances in gene therapy and curative treatments, such as hematopoietic stem cell transplantation, offer potential avenues for addressing the underlying genetic defect and preventing organ damage in the future^5–8^.

## Therapeutic strategies

### Gardos channel inhibitors

Gardos channel inhibitors represent a promising class of therapeutic agents for the management of sickle cell anemia (SCA)^33^. These inhibitors target the Gardos channel, also known as the calcium-activated potassium channel (KCa3.1), which plays a crucial role in regulating potassium efflux and cell dehydration in red blood cells (RBCs). Understanding the significance of Gardos channel inhibitors involves exploring their mechanism of action, clinical implications, and potential benefits for patients with SCA. The Gardos channel is activated by intracellular calcium (Ca2+) and facilitates the efflux of potassium ions (K+) from RBCs. This process is linked to the dehydration of RBCs, a critical factor in the pathophysiology of SCA. Increased potassium efflux leads to cell shrinkage and higher hemoglobin S (HbS) concentration, promoting the polymerization of HbS and the formation of sickle-shaped cells. By inhibiting the Gardos channel, potassium efflux is reduced, which helps maintain RBC hydration and decreases the propensity of cells to sickle under conditions of hypoxia or stress.

Gardos channel inhibitors, such as senicapoc (ICA-17043), have been studied for their ability to reduce RBC dehydration in individuals with SCA^34^. By inhibiting potassium efflux, these inhibitors help preserve RBC volume and reduce the concentration of intracellular HbS. This effect potentially lowers the incidence of vaso-occlusive crises (VOC) and other complications associated with SCA, improving overall patient outcomes. One of the primary benefits of Gardos channel inhibitors is their potential to mitigate the frequency and severity of VOC, which are characterized by intense pain episodes due to microvascular occlusions by sickled RBCs. By maintaining RBC hydration and reducing sickling, these inhibitors may decrease the need for hospitalizations and emergency treatments related to VOC, thereby enhancing the quality of life for patients with SCA. In addition to symptomatic relief, Gardos channel inhibitors hold promise as disease-modifying agents in SCA^1^. By targeting a key mechanism involved in the pathogenesis of the disease—RBC dehydration and sickling—these inhibitors may slow disease progression and prevent long-term complications, such as organ damage and chronic anemia. Long-term studies are needed to fully evaluate their impact on disease course and patient outcomes.

### Hydration therapy

Hydration therapy plays a crucial role in the management of various medical conditions, including sickle cell anemia (SCA)^35^. In the context of SCA, hydration therapy focuses on maintaining adequate fluid intake to optimize red blood cell (RBC) hydration, reduce the viscosity of blood, and mitigate the complications associated with RBC sickling and dehydration. Understanding the principles, benefits, and clinical applications of hydration therapy in SCA is essential for effectively managing this complex genetic disorder. Individuals with SCA are prone to chronic dehydration due to increased fluid loss through various mechanisms, including increased urine output and insensible losses. Hydration therapy aims to counteract these losses by ensuring adequate fluid intake, typically through oral hydration with water and electrolyte-rich fluids. Dehydration in SCA leads to increased blood viscosity, exacerbating vaso-occlusive crises (VOC) and impairing blood flow to vital organs^5^. Adequate hydration helps lower blood viscosity, promoting smoother blood circulation and reducing the likelihood of RBC sickling and vascular occlusions. Dehydrated RBCs in SCA have higher concentrations of hemoglobin S (HbS), which increases the risk of polymerization and sickling under hypoxic conditions. Hydration therapy helps maintain RBC volume and reduces intracellular HbS concentration, thereby minimizing sickling and its associated complications.

Hydration therapy is integral to the acute management of VOC in SCA. During VOC episodes, increased fluid intake helps improve blood flow, alleviate pain, and prevent further sickling of RBCs. Intravenous hydration may be necessary for patients experiencing severe VOC to rapidly restore fluid balance and hydration status. Chronic dehydration in SCA contributes to various complications, including renal dysfunction, pulmonary complications (e.g. acute chest syndrome), and impaired cognitive function. Hydration therapy plays a preventive role by reducing the frequency and severity of these complications, enhancing overall health outcomes for individuals with SCA. In addition to acute crises, hydration therapy forms an essential component of the long-term management of SCA. Patients are encouraged to maintain adequate hydration throughout their daily routines to prevent dehydration-related complications and support overall well-being. Monitoring fluid intake and urine output, particularly during periods of illness or increased physical activity, helps optimize hydration therapy effectiveness. Encouraging individuals with SCA to drink sufficient fluids throughout the day, including water and electrolyte-containing beverages, is fundamental. Educating patients and caregivers about the importance of hydration and providing practical guidance on fluid intake goals can help maintain hydration status. In severe cases of VOC or when oral intake is insufficient, intravenous hydration with isotonic solutions (e.g. normal saline) may be administered under medical supervision. This approach rapidly restores fluid balance and electrolyte levels, supporting the resolution of acute crises^13–15^.

### Comprehensive management

Comprehensive management of sickle cell anemia (SCA) involves a multifaceted approach aimed at addressing the diverse clinical manifestations and complications associated with this genetic disorder^36^. The goal is to improve quality of life, prevent acute complications, and mitigate long-term organ damage. Hydroxyurea is a cornerstone of disease-modifying therapy for SCA. It works by increasing fetal hemoglobin (HbF) production, which inhibits the polymerization of sickle hemoglobin (HbS) and reduces the frequency of vaso-occlusive crises (VOC). Hydroxyurea has been shown to decrease pain episodes, acute chest syndrome, and the need for blood transfusions in patients with SCA. Emerging therapies, such as gene therapy and gene editing techniques, hold promise for correcting the underlying genetic defect responsible for SCA. These approaches aim to provide a potential cure by restoring normal hemoglobin production and preventing sickle cell formation. Pain is a hallmark symptom of SCA, primarily due to VOC and chronic pain syndromes^37^. Effective pain management involves a combination of nonopioid analgesics, opioids for severe pain episodes, and non-pharmacological approaches such as heat therapy and relaxation techniques. Maintaining adequate hydration is critical for preventing RBC sickling and reducing the viscosity of blood. Hydration therapy includes encouraging oral fluid intake and, in severe cases, administering intravenous fluids during VOC episodes or periods of increased fluid needs. Due to functional asplenia in many patients with SCA, antibiotic prophylaxis (e.g. penicillin) and vaccinations against encapsulated bacteria (e.g. pneumococcus, Haemophilus influenzae type b, and meningococcus) are essential to prevent serious infections, particularly in children. Chronic transfusion therapy may be indicated for patients with severe SCA complications, such as stroke prevention in high-risk individuals or the management of severe anemia. Regular transfusions help dilute sickle cells and decrease the risk of VOC.

Acute chest syndrome (ACS) is a life-threatening complication characterized by pulmonary vaso-occlusion and inflammation. Management involves prompt recognition, supportive care with oxygen therapy and antibiotics, and sometimes transfusion therapy to improve oxygenation. Stroke prevention is crucial in SCA due to the increased risk of cerebral infarctions^38^. Transcranial Doppler (TCD) screening identifies children at high-risk for stroke, who may benefit from chronic transfusion therapy or other interventions to reduce stroke risk. Sickle cell nephropathy can lead to chronic kidney disease (CKD) due to microvascular occlusions and chronic hemolysis. Management includes monitoring renal function, controlling hypertension, and addressing iron overload to preserve kidney function. Cardiac complications, such as pulmonary hypertension and heart failure, require comprehensive cardiovascular monitoring and management. This includes regular echocardiography, medications to manage pulmonary hypertension, and iron chelation therapy to prevent iron overload cardiomyopathy. Educating patients and caregivers about SCA, including symptoms, complications, and the importance of adherence to treatment regimens, is essential^39^. Empowering patients with knowledge enhances self-management and improves treatment outcomes. Living with a chronic illness like SCA can impact mental health and quality of life. Psychosocial support, including counseling, support groups, and social services, helps patients and families cope with the emotional and social challenges associated with the disease. Regular monitoring of clinical and laboratory parameters is crucial in SCA management to assess treatment efficacy, detect complications early, and adjust therapies as needed. This includes monitoring hemoglobin levels, reticulocyte count, kidney function, iron status, and neurocognitive function.

## Results

SCA is caused by a point mutation in the β-globin gene, leading to the production of abnormal hemoglobin S (HbS). Under conditions of low oxygen tension, HbS molecules polymerize, causing red blood cells (RBCs) to assume a sickle shape. This polymerization process is central to the pathogenesis of SCA, contributing to RBC rigidity, reduced deformability, and increased susceptibility to hemolysis. The review highlighted that polymerized HbS promotes RBC adhesion to vascular endothelium, initiating vaso-occlusion in small blood vessels. This process obstructs blood flow, leading to tissue ischemia and acute pain crises characteristic of SCA. Endothelial dysfunction, inflammatory mediators, and adhesive interactions between sickled RBCs and endothelial cells further exacerbate Vaso-Occlusive Crises (VOC), perpetuating tissue damage and organ dysfunction. Chronic hemolysis in SCA results from the fragility of sickled RBCs and their shortened lifespan. Released hemoglobin leads to scavenging of nitric oxide (NO), impairing vasodilation and promoting vasoconstriction. Dysregulation of ion transport systems, including increased potassium efflux via the Gardos channel (KCa3.1), contributes to RBC dehydration and further promotes sickling. The review highlighted that chronic inflammation in SCA is driven by activated leukocytes, endothelial activation, and the release of proinflammatory cytokines and adhesion molecules. This inflammatory milieu contributes to a prothrombotic state, endothelial dysfunction, and tissue injury, exacerbating the pathophysiology of the disease.

## Discussion

Targeting the molecular pathways involved in HbS polymerization, such as modifying the balance of HbF production or developing agents that inhibit polymer formation, represents promising therapeutic strategies^40^. Additionally, interventions aimed at reducing chronic hemolysis, preserving RBC hydration, and mitigating inflammatory responses may alleviate symptoms and prevent complications in patients with SCA. Insights into the pathophysiological mechanisms of SCA inform clinical management strategies aimed at preventing vaso-occlusive crises, managing chronic pain, and reducing organ damage. Therapeutic approaches may include hydroxyurea to increase HbF levels, blood transfusions to dilute sickled cells, and novel agents targeting specific molecular pathways involved in RBC sickling and adhesion. Advances in gene therapy and gene editing technologies offer potential curative approaches by correcting the genetic defect responsible for SCA^41^. Ongoing research focuses on optimizing these therapies, addressing challenges such as delivery methods and long-term efficacy, to pave the way for personalized medicine in the treatment of SCA. The review highlights the importance of multidisciplinary care and comprehensive management strategies for individuals with SCA. This includes genetic counseling, early detection of complications through regular monitoring, and addressing psychosocial needs to improve overall patient outcomes and quality of life.

### Recommendations

Based on the findings and discussion of the pathophysiological insights into sickle cell anemia (SCA), several recommendations can be made to guide future research and clinical practice:

1. Enhanced understanding of molecular pathways: Further research should focus on elucidating the intricate molecular pathways involved in hemoglobin polymerization, RBC sickling, and vaso-occlusive crises (VOC) in SCA. This includes investigating novel therapeutic targets that can interrupt these pathways to prevent or mitigate disease progression.
2. Development of targeted therapies: There is a critical need to advance the development of targeted therapies for SCA based on the identified pathophysiological mechanisms. This includes optimizing existing treatments like hydroxyurea and exploring new pharmacological agents, gene therapies, and gene editing technologies aimed at correcting the underlying genetic defect or modulating disease severity.
3. Early intervention and comprehensive management: Emphasize the importance of early intervention and comprehensive management strategies in SCA to prevent complications and improve patient outcomes. This involves regular monitoring of clinical and laboratory parameters, timely initiation of disease-modifying therapies, and personalized care plans tailored to individual patient needs.
4. Integration of psychosocial support: Incorporate psychosocial support services into routine care for individuals with SCA to address the emotional, social, and educational challenges associated with chronic illness. This includes providing counseling, support groups, and educational resources to empower patients and caregivers in managing the disease effectively.
5. Advancement in genetic counseling and screening programs: Expand genetic counseling services and promote broader implementation of newborn screening programs for SCA to facilitate early diagnosis, genetic education, and timely intervention. Early identification of at-risk individuals allows for proactive management and reduces the burden of acute complications.
6. Healthcare provider education and training: Enhance education and training for healthcare providers to improve awareness, knowledge, and skills in managing SCA. This includes fostering interdisciplinary collaboration among hematologists, primary care physicians, genetic counselors, and allied health professionals to deliver coordinated and holistic care.
7. Patient advocacy and community engagement: Foster patient advocacy efforts and community engagement initiatives to raise awareness about SCA, reduce stigma, and advocate for equitable access to healthcare resources and supportive services. Empowering patients and families through education and advocacy enhances disease management and promotes better health outcomes.
8. Promotion of research collaborations: Encourage collaborative research efforts among academic institutions, healthcare providers, industry partners, and patient advocacy organizations to accelerate the translation of scientific discoveries into clinical practice. Collaborative initiatives can facilitate the development of innovative therapies and personalized treatment approaches for SCA.

## Conclusion

The management of sickle cell anemia (SCA) is a multifaceted endeavor that encompasses a range of therapeutic strategies aimed at alleviating symptoms, preventing complications, and improving overall quality of life for individuals affected by this genetic disorder. Through a comprehensive approach that integrates disease-modifying therapies like hydroxyurea, supportive care measures such as pain management and hydration therapy, and proactive management of complications like vaso-occlusive crises and organ damage, healthcare providers can significantly enhance patient outcomes. The advent of novel therapies, including gene editing and gene therapy approaches, holds promise for potentially curing SCA by addressing the underlying genetic defect responsible for abnormal hemoglobin production. These advancements underscore ongoing efforts to transform the treatment landscape and offer hope for a future where individuals with SCA can live healthier lives, free from the burden of chronic pain and complications. Moreover, patient education and psychosocial support play pivotal roles in empowering individuals and families to manage SCA effectively. Educating patients about the disease, promoting adherence to treatment regimens, and providing emotional support are essential components of holistic care that promote self-management and improve overall well-being.

## Ethical approval

Not applicable.

## Consent

Not applicable.

## Source of funding

Not applicable.

## Author contribution

Emmanuel performed the following roles: conceptualisation, methodology, supervision, draft writing, editing, and approval before submission.

## Conflicts of interest disclosure

The author declares no conflict of interest.

## Research registration unique identifying number (UIN)

Not applicable.

## Guarantor

Emmanuel Ifeanyi Obeagu.

## Data availability statement

Not applicable.

## Provenance and peer review

Not applicable.
